# Stabilization of the CD81 Large Extracellular Loop with *De Novo* Disulfide Bonds Improves Its Amenability for Peptide Grafting

**DOI:** 10.3390/pharmaceutics10030138

**Published:** 2018-08-27

**Authors:** Stefan Vogt, Gerhard Stadlmayr, Katharina Stadlbauer, Flávio Sádio, Peter Andorfer, Johannes Grillari, Florian Rüker, Gordana Wozniak-Knopp

**Affiliations:** 1 acib GmbH (Austrian Centre of Industrial Biotechnology), Petersgasse 14, A-8010 Graz, Austria; stefan.vogt@boku.ac.at (S.V.); florian.rueker@boku.ac.at (F.R.); 2 Christian Doppler Laboratory for Innovative Immunotherapeutics, Department of Biotechnology, University of Natural Resources and Life Sciences (BOKU), Vienna, Muthgasse 18, 1190 Vienna, Austria; gerhard.stadlmayr@boku.ac.at (G.S.); katharina.stadlbauer@boku.ac.at (K.S.); flavio.sadio@boku.ac.at (F.S.); peter.andorfer@boku.ac.at (P.A.); 3 Christian Doppler Laboratory for Biotechnology of Skin Aging, Department of Biotechnology, University of Natural Resources and Life Sciences (BOKU), Vienna, Muthgasse 18, 1190 Vienna, Austria; johannes.grillari@boku.ac.at; 4 Evercyte GmbH, Muthgasse 18, 1190 Wien, Austria

**Keywords:** CD81 large extracellular loop, peptide grafting, antigen recognition unit, stability engineering

## Abstract

Tetraspan proteins are significantly enriched in the membranes of exosomal vesicles (EVs) and their extracellular domains are attractive targets for engineering towards specific antigen recognition units. To enhance the tolerance of a tetraspanin fold to modification, we achieved significant thermal stabilization of the human CD81 large extracellular loop (hCD81 LEL) via *de novo* disulfide bonds. The best mutants were shown to exhibit a positive shift in the melting temperature (*T*_m_) of up to 25 °C. The combination of two most potent disulfide bonds connecting different strands of the protein resulted in a mutant with a *T*_m_ of 109 °C, 43 °C over the *T*_m_ of the wild-type hCD81 LEL. A peptide sequence binding to the human transferrin receptor (hTfr) was engrafted into the D-segment of the hCD81 LEL, resulting in a mutant that still exhibited a compact fold. Grafting of the same peptide sequence between helices A and B resulted in a molecule with an aberrant profile in size exclusion chromatography (SEC), which could be improved by a *de novo* cysteine bond connecting both helices. Both peptide-grafted proteins showed an enhanced internalization into the cell line SK-BR3, which strongly overexpresses hTfr. In summary, the tetraspan LEL fold could be stabilized to enhance its amenability for engineering into a more versatile protein scaffold.

## 1. Introduction

In the last decade, the research into exosomes has intensified as they have been recognized as significant mediators of cell-to-cell communication [1]. Exosomes are released from multivesicular bodies upon their fusion with the plasma membrane, and released vesicles function as delivery vehicles transferring functional RNAs, exosomal DNA, and transmembrane proteins including receptors to cells in the surrounding environment [2]. Regarding their advantages as a potential therapeutic moiety, including favorable properties such as low immunogenicity and low cytotoxicity, the interest in their application has triggered the development of new methods for encapsulation, improved cytosolic release of exosomal contents, enhanced cellular uptake, and more specific cellular targeting [3]. Vesicular uptake of exosomes is cell type-specific and can involve membrane fusion or endocytosis, and can even be induced by stimulation of oncogenic cancer receptors [4]. To achieve tissue specific delivery, targeting of exosomes can be optimized by engineering the source cells to overexpress exosomal membrane proteins such as tetraspanins, harboring receptor-specific ligand peptides as recognition units [5]. Tetraspanins are known as molecular facilitators, associating in large cell-signaling complexes known as the tetraspan web, which involves members of other protein families such as integrins and coreceptor molecules. Furthermore, such large membrane protein assemblies may be associated with lipid rafts [6,7]. The functions of tetraspanins CD9, CD63, and CD81 as ligands for the endocytosis of exosomes have been reported [8,9,10]; however the mechanism of uptake has not yet been clarified. 

In spite of its almost ubiquitous distribution, tetraspan protein CD81 is the major protein enriched in the exosomal fraction of multivesicular bodies [11]. The large extracellular loop of CD81, topologically located between transmembrane domains 3 and 4, is characterized by five helical elements forming a mushroom-like structure [12,13] stabilized by two pairs of cysteines. This motif is conserved among the protein members of the tetraspanin family [14] and the oxidation of cysteine bonds is a prerequisite for high-affinity binding of the E2 envelope protein of the hepatitis C virus (HCV), the natural ligand of CD81 [15]. Correct pairing of the cysteines is also crucial for recognition by the antibody M38 [16], which does not bind to denatured or reduced proteins but can react with the membrane-bound human CD81 (hCD81) as well as a native form of purified soluble hCD81. The crystal structure of the human CD81 large extracellular loop (hCD81 LEL), solved at 1.6 Å, revealed a new type of protein fold [12], and a subsequent sequence analysis of 160 tetraspanin family members indicated that their fold and key structural features are conserved [17]. Apart from cysteine bridges, hCD81 LEL is stabilized by the invariant residues Gly157 and Pro176, which are located to accommodate cysteine connections as well as Tyr127, which is fully buried and contributes to the hydrogen bonding network together with His151 and Cys190. Soluble hCD81 LEL assembles into dimers around a two-fold axis, and the contact between the protomers is in a low-polarity region between the helices of each interacting partner and between helix B and C-terminal residues of the opposite protomer. The N- and C-termini of the protomers fall in the central region on opposite faces of the assembled dimer, similar to the dimeric assembly at the cell surface where transmembrane segments are also present. A second low-polarity region comprises the solvent-exposed surface of helices C and D, which is energetically unfavorable. According to the solution studies, helix D is fairly unstructured and attains helical conformation only upon binding with certain antigens [18]. The sequence alignments of the tetraspanin family members indeed show an increased variability in this region, including insertions and deletions [19]. It has been suggested that this surface area might be involved in a species- or tetraspanin-specific recognition process [20], which could hint to the possibility of heterodimeric tetraspanin species assembly [21]. In particular, segment D of CD81 should be able to guide specific homomeric clustering [22].

To enhance their potential as the next-generation therapeutic carriers, exosome-mediated delivery systems need to be further developed, especially to improve their inherently low efficiency of cellular uptake, which can be achieved by the engineering of exosomal membrane proteins. In our approach, we attempted to improve the biophysical properties of hCD81 LEL, aiming towards its functionalization as a small ligand recognition unit. The expression level of the wild-type his-tagged soluble protein in a mammalian expression system in standard laboratory small-scale conditions was high enough to allow detailed characterization for its dimeric status and thermostability. First, we introduced *de novo* pairs of cysteine residues at different regions of the molecule to increase its thermostability, which should allow extensive modifications of the amino acid sequence that may be required to achieve specific antigen recognition. Further, we modified the C-terminal part of the helix D in the hCD81 LEL by introducing a human transferrin receptor (hTfr)-specific peptide, which can recognize hTfr with an affinity of 10^−7^ M [23]. Next, the short hairpin loop between helices A and B was targeted for mutagenesis to introduce the same peptide sequence at this alternative site. A stabilizing disulfide bond connecting helices A and B was shown to enhance the biophysical properties of the protein modified in this way.

## 2. Materials and Methods 

### 2.1. Molecular Modeling 

DSDBASE (http://caps.ncbs.res.in/dsdbase/dsdbase.html) [24] was used as a prediction tool for the identification of positions with the potential to harbor cysteine residues suitable for the creation of intradomain disulfide bonds. The algorithm was used to analyze the hCD81 LEL crystal structure 1G8Q [14] for the distances between Cα and Cβ atoms of neighboring amino acid residues as well as for torsion angles and resulting S-S bond lengths. Out of 36 predicted possible disulfide bonds we selected 11 that seemed to be the ones with the highest likelihood of success as judged by visual examination of the crystal structure. Five of those were predicted by the DSDBASE program both in protomer A and protomer B of the hCD81 LEL.

Molecular models of hCD81 LEL mutants engrafted with hTfr-targeting peptides were constructed using the homology modeling server SWISS-MODEL (https://swissmodel.expasy.org/) [25,26]. Sequences of the constructs are listed in Supplementary Table S1.

### 2.2. Production of Recombinant Proteins

The fragment encoding hCD81 LEL (fragment Phe113-Lys201) (numbering according to Protein Data Base (PDB) entry 1G8Q) was amplified from a synthetic construct with the full-length CD81 sequence (Geneart, Regensburg, Germany). Mutagenesis to introduce grafted peptide sequences as well as mutagenesis of single chosen amino acid residues to cysteine was performed using QuikChange Lightning Mutagenesis kit (Agilent, Santa Clara, CA, USA), exactly according to manufacturer’s instructions with oligonucleotides listed in Supplementary Table S2. hCD81 LEL variants were cloned into the pTT22SSP4 mammalian expression vector (CNRC, Ottawa, ON, Canada) and expressed in two different expression systems. For prescreening, the constructs were expressed in HEK293-6E cells (CNRC) at a 2-mL-scale in F17 medium supplemented with 4 mM glutamine and 50 µg/mL G-418 on an orbital shaker at 180 rpm, at 37 °C under 5% CO_2_ for 4 days, with feeding of TN-20 to an end concentration of 0.8% on the second day after transfection. Mutants selected for further characterization were transfected into ExpiCHO cells (Thermo Fisher, Waltham, MA, USA) exactly according to manufacturer’s instructions. Cultivation of the cells proceeded according to the MaxTiter protocol. Supernatants were harvested after 14 days and purified using nickel-nitrilotriacetic acid (Ni-NTA) affinity chromatography. After clarification, the samples were buffered with phosphate-buffered saline (PBS, Boston, MA, USA) with 20 mM imidazole and pH 7.5, and passed over an Excel Ni-NTA column (GE Healthcare, Chicago, IL, USA) equilibrated with the same buffer. The his-tagged hCD81 LEL was eluted with a gradient from 20 to 500 of mM imidazole in five column volumes. Fractions containing the target protein were pooled and dialyzed twice against the 100-fold volume of PBS overnight at 4 °C. The proteins were stored at −80 °C until use.

### 2.3. Biophysical Characterization of the Human CD81 Large Extracellular Loop (hCD81 LEL) Mutants

#### 2.3.1. SDS-PAGE

Two µg of purified protein preparations were mixed with loading sample buffer and resolved on 4–12% Novex NuPAGE gels, run in MES buffer, stained with NovexBlue staining kit (Thermo Fisher, Waltham, MA, USA), and destained with distilled water.

#### 2.3.2. Size Exclusion Chromatography (SEC)-High Press ure Liquid Chromatography (HPLC) and Multi-Angle Light Scattering (MALS)

A LC-20A Prominence system (Shimadzu, Kyoto, Japan) equipped with a diode array detector and a refractive index detector was used to perform SEC-HPLC with a Superdex 200 Increase 10/300 GL column (GE Healthcare, Chicago, IL, USA). The mobile-phase buffer used was PBS with 200 mM NaCl. Chromatography was conducted with a constant flow rate of 0.75 mL/min. In total, 200 µg of protein at about 2 mg/mL were loaded on the column for analysis. Column calibration was performed with a set of molecular weight standards ranging from 10 to 500 kDa (Bio-Rad, Hercules, CA, USA). In-line multi-angle light scattering was analyzed on a miniDAWN TREOS II MALS apparatus (Wyatt Technologies, Santa Barbara, CA, USA). The defined chromatographic peak of the protein was used to calculate its molecular mass using ASTRA software, version 6.1 (Wyatt Technologies, Dernbach, Germany).

#### 2.3.3. Differential Scanning Chromatography (DSC)

DSC experiments were performed on MicroCal PEAQ-DSC Automated system (Malvern, Malvern, UK), using an 80-µM protein solution diluted in PBS at pH 7.4. The heating was performed from 20 °C to 110 °C at a rate of 1 °C/min. Protein solution was then cooled in situ and an identical thermal scan was run to obtain the baseline for subtraction from the first scan. All measurements were taken in duplicates. Fitting was performed with MicroCal PEAQ-DSC Software using the non-two-state transition mechanism.

#### 2.3.4. Circular Dichroism (CD) Spectroscopy 

The far-UV CD spectra were measured on a Chirascan spectropolarimeter (Applied Photophysics, Surrey, UK) at 25 °C using a 1-mm quartz cuvette. The protein preparations were diluted in PBS to 200 µg/mL. The CD spectra of the buffer solutions were subtracted from the sample spectra before conversion to CD absolute units.

### 2.4. ELISA to Detect the Reactivity with M38 Antibody

ELISA plates (Maxisorp, NUNC, Roskilde, Denmark) were coated with an anti-hCD81 M38 antibody (Thermo Fisher) at 5 µg/mL in PBS for 1 h at room temperature (RT). After blocking with 5% bovine serum albumin (BSA)-PBS for 1 h at RT, supernatants of HEK293-6E cells transfected with hCD81 LEL variants or purified variants of hCD81 LEL diluted in 2.5% BSA-PBS were allowed to bind for 1 h at RT. After extensive washing, the binding of mutant proteins was detected with an anti-his-horseradish peroxidase (HRP) conjugated antibody (QIAgen, Hilden, Germany), diluted 1:2500 in 2.5% BSA-PBS. Antibody binding was revealed with 3,3′,5,5′-tetramethylbenzidine (TMB) (Sigma Aldrich, St. Louis, MO, USA); the reaction was stopped by adding an equal volume of 30% H_2_SO_4_ and absorbance was read at 450/620 nm.

### 2.5. Flow Cytometry

SK-BR3 cells (ATCC HTB-30) were cultured in DMEM with 10% FCS and penicillin–streptomycin in humidified atmosphere at 37 °C under 5% CO_2_. For a fluorescence-activated cell sorting (FACS) experiment confirming the high expression of hTfr on SK-BR3 cells, cells were harvested and blocked in 2% BSA-PBS for 30 min on ice. Then, 100,000 cells/well of a 96-well plate were stained with three-fold serial dilution of anti-hTfr MEM-75 antibody in 2% BSA-PBS for 30 min on ice. Dead cells were excluded from the measurement by staining with 7-aminoactinomycin D (7-AAD) and mean fluorescence intensity (MFI) values of the live cell population were recorded. After a brief wash in ice-cold PBS, a 1:200 dilution of anti-mouse (Fab’)_2_–FITC conjugate (Sigma-Aldrich, St. Louis, MO, USA) as a secondary reagent in 2% BSA-PBS was used to detect test protein binding. Determination of the copy number of hTfr was performed with QIFIKIT^®^ (Agilent, Santa Clara, CA, USA), exactly according to manufacturer’s instructions. Briefly, five populations of calibration beads coated with graded numbers of mouse mAb molecules were used as a calibration standard. Then, 100,000 cells/well were labeled with primary mouse MEM-75 antibody at a saturation concentration of 10 nM. Beads and cells were then stained with a secondary goat anti-mouse (Fab′)_2_–FITC conjugate (Agilent, Santa Clara, CA, USA) and their fluorescence determined using Gallios flow cytometer (Beckman Coulter, Brea, CA, USA). hTfr cell surface levels were calculated using a calibration line for fluorescence of beads versus the bead surface coating level.

### 2.6. Receptor-Mediated Internalization into the Target Cell Line

Protein preparations were labeled with NHS-Alexafluor488 conjugate (Thermo Fisher, Waltham, MA, USA) and allowed to internalize into SK-BR3 cells (ATCC^®^ HTB-30^™^) that were seeded into 6-well plates at 1 × 10^6^ cells/well overnight, in triplicates for one read. After 12 h of exposure to the 10-µM protein preparation, cells were harvested with a cell scraper, blocked with 2% BSA–PBS, and incubated with 500 µg/mL Alexafluor488-quenching antibody (Thermo Fisher, Waltham, MA, USA) for 30 min on ice. Median fluorescence of the population was determined for the quenched and the non-quenched sample after excluding the dead cells labeled with 7-AAD. 

## 3. Results

### 3.1. Expression and Purification of the Wild-Type Protein

**Figure 1:**
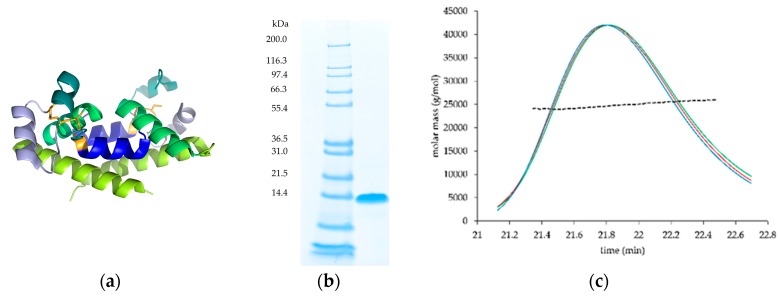
The wild-type human CD81 large extracellular loop (hCD81 LEL). (**a**) Crystal structure of hCD81 LEL (PDB 1G8Q). Lemon: helix A; green: helix B; light blue: helix C; teal: helix D; deep blue: helix E; orange: native cysteine bridges. The figure was prepared with PyMOL (PyMOL Molecular Graphics System, Version 1.3 Schrödinger, LLC); (**b**) SDS-PAGE of purified hCD81 LEL; (**c**) Multi-angle light scattering (MALS) analysis of purified hCD81 LEL. Black dashed line: molar mass; red: light scattering detector trace; green: UV detector trace; blue: refractory index detector trace.

The hCD81 LEL (crystal structure 1G8Q with highlighted structural subunits in Figure 1a) was purified at 140 mg/L from ExpiCHO supernatant using single-step HisTrap chromatography. SDS-PAGE of the purified protein revealed a single band of the molecular size that corresponds to a monomeric species (Figure 1b); however, SEC analysis under native conditions suggested a dimeric form of the protein, which was confirmed by MALS analysis (Figure 1c). The molecular weight determined with MALS suggested 24.11 kDa, which corresponded well to the theoretical molecular mass of 24.354 kDa. Dimerization of recombinantly produced hCD81 has been observed before [27,28] and was also reported for other members of the tetraspanin family [27].

### 3.2. Design of *De Novo* Disulfide Bonds and Preliminary Screening of Candidate Mutants for Expression

**Figure 2:**
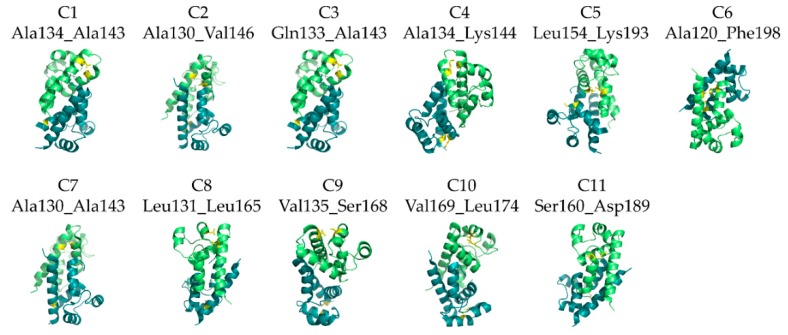
Cartoon diagrams of proposed hCD81 LEL mutants with pairs of amino acids mutated to cysteine indicated in yellow. Green: protomer A; teal: protomer B. Figure was prepared with PyMOL (PyMOL Molecular Graphics System, Version 1.3 Schrödinger, LLC).

By analysis of the hCD81 LEL crystal structure (PDB 1G8Q), 36 mutants that might be stabilized by a novel cysteine bond were proposed by the DSDBASE algorithm. As the number of endorsed residues is theoretically decisive of the free energy change in respect to the unmutated protein [29], the proposed candidate positions for mutagenesis were examined by visual inspection of the crystal structure (Figure 2).

In five of the proposed mutants, the *de novo* cysteine bond was placed to connect helices A and B (Table 1). In two of the mutants, helix A connected with helix C. Another proposed disulfide bond would connect helices B and E; in one mutant the loop structure N-terminally to helix C would connect with the start of segment D, and in one mutant almost the entire hCD81 LEL would span between newly introduced cysteines connecting helices A and E. Finally, in one of the mutants the novel cysteines, both positioned in segment C, would span a loop of only few residues. All of these mutants were cloned into a mammalian expression vector and expressed in HEK293-6E at a 2-mL scale. Nine mutants expressed at a level that was equal or superior to the wild type. One of the mutants (C5, Leu154Cys/Lys193Cys) was not expressed at all, one (C6, Ala120Cys/Phe198Cys) was expressed at a level of about 10% of the wild-type protein, and one (C10, Val169Cys/Leu174Cys) formed a conspicuous dimer; therefore, they were excluded from further analysis. Supernatants containing mutant proteins were tested for the reactivity with the conformation-reporter antibody M38 [16,30] in ELISA and their binding was detected with an anti-his-tag reactive antibody (Supplementary Figure S1). All but one (C8, Leu131Cys/Leu165Cys) of the expressed proteins were detected, indicating that the pairing of the native cysteines proceeded in a correct way. The exchange of residues Leu131 and Leu165 for cysteines in the non-reactive mutant C8 either disturbed the formation of the native cysteine bonds or altered the conformation of segment D, the reported epitope for structural-dependent binding of the M38 antibody [15].

### 3.3. Expression and Characterization of the Cysteine-Stabilized Mutants

**Figure 3:**
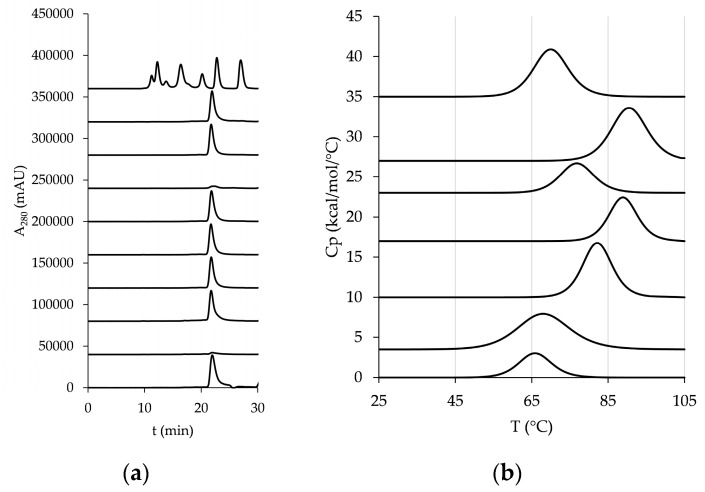
Significant thermal stabilization of hCD81 LEL. (**a**) Size Exclusion Chromatography-High Press ure Liquid Chromatography (SEC-HPLC) profiles: from bottom to top: wild-type hCD81 LEL, C1, C2, C3, C4, C7, C8, C9, C11 and molecular weight standards at 670, 158, 44, 17, and 1.3 kDa (Bio-Rad); (**b**) DSC profiles of (from bottom to top): wild-type hCD81 LEL, C2, C3, C4, C7, C9, and C11.

The candidate stabilization mutants were subsequently expressed in the ExpiCHO expression system and purified using Ni-NTA affinity chromatography (Supplementary Figure S2). For all eight investigated mutants, yields similar or superior to the wild type were achieved. Purified proteins were analyzed with Size Exclusion Chromatography (SEC)-High Pressure Liquid Chromatography (HPLC) (SEC-HPLC) in native conditions, and with the exception of C1 and C8, all exhibited a sharp well-resolved peak with elution at a time corresponding to the wild-type hCD81 LEL (Figure 3a). Thermal unfolding of the hCD81 LEL mutants proceeded in a single transition as assessed with DSC (Figure 3b).

The *T*_m_ of the wild-type mutant was determined to be at 66.15 ± 0.25 °C, with an onset at 51.45 ± 0.75 °C and completion at 82.25 ± 0.25 °C. Six of eight tested mutants showed an increase in the *T*_m_, the most stable one being C9 (Val135Cys/Ser168Cys), shifting to a *T*_m_ of 90.6 °C. Several mutants that were designed to interconnect helix A and helix B exhibited different levels of improvement in their thermostability with respect to the wild-type protein, with the most potent C4 (Ala134Cys/Lys144Cys) reaching a *T*_m_ of 88.95 ± 0.05 °C. Interestingly, the strongly stabilized mutants also showed a significantly improved expression level with respect to the wild-type hCD81 LEL: an about a three-fold yield was achieved for C4 and C9. No reliable endotherm was recorded for the mutants Ala134Cys/Ala143Cys and Leu131Cys/Leu165Cys, indicating that the molar heat capacity of these proteins was decreased in respect to the wild-type molecule. Surprisingly, the novel disulfide bond in C1 (Ala134Cys/Ala143Cys) is positioned similarly as in most potent stabilizing mutant C4, but nevertheless resulted in a hCD81 LEL mutant with an aberrant HPLC profile, indicating probably an undesired structural constraint exerted on the protein fold. Such phenomena could be explained with use of modeling approaches that do not only depend on rigid body structural parameters but take into account the dynamics of atomic-level phenomena using molecular dynamics simulation.

The mutant C2 (Ala130Cys/Val146Cys) was improved in the *T*_m_ only by 1.2 °C compared to the wild-type hCD81 LEL; however it differed from the wild-type protein and all other stabilized mutants in that it could refold reversibly when cooled in situ after it had been heated up to 110 °C. For the evaluation of this DSC experiment the endotherm recorded with PBS was used as a baseline for subtraction instead of that of the denatured protein solution.

**Figure 4:**
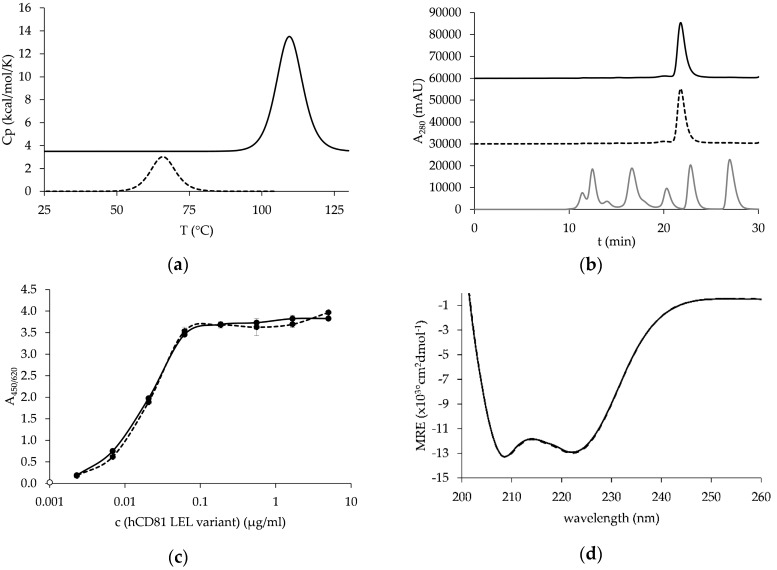
Characterization of hCD81 LEL_C4C9 mutant (full line) and comparison to wild-type hCD81 LEL (dashed line) with: (**a**) DSC, (**b**) SEC in native conditions (trace of the Bio-Rad molecular weight marker is indicated with a gray line and peaks correspond to 670, 158, 44, 17 and 1.3 kDa), (**c**) reactivity with M38 antibody in ELISA (signal from secondary reagent is indicated with an empty circle), and (**d**) far UV-CD.

A hCD81 LEL mutant with a combination of potently stabilizing novel disulfide bonds Ala134Cys/Lys144Cys and Val135Cys/Ser168Cys was then produced and tested for its thermostability. Thermal unfolding was recorded up to 130 °C and proceeded in a single event at a *T*_m_ of 109.40 ± 0.25 °C (Figure 4a), which is 43 °C above that of the wild-type hCD81 LEL. This protein termed C4C9 migrated in SEC in native conditions as a single sharp peak at a time characteristic for the wild-type protein (Figure 4b). Importantly, the stabilized mutant was able to bind to the structure-reporter antibody M38 with the same extent as wild-type hCD81 LEL (Figure 4c). The far-UV CD spectrum for this mutant was examined and found to be identical to the one obtained for wild-type hCD81 LEL, and was typical of a protein with high α-helix content with two characteristic minima at 208 and 222 nm (Figure 4d), similarly to previously published results [18]. 

### 3.4. Peptide Grafting

**Figure 5:**
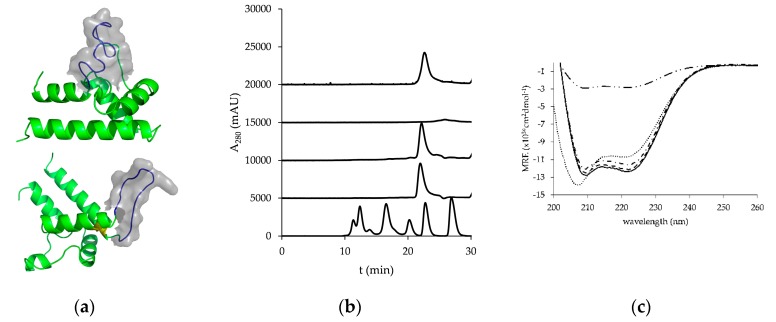
hCD81 LEL derivatized with anti-hTfr peptide: (**a**) Cartoon models of hCD81 LEL engrafted with anti-hTfr peptide (blue) in the D-segment (upper) or in the loop connecting helices A and B (lower). De novo introduced cysteine residues in hCD81 LEL_Tfr2_C4 are in yellow. Amino acid residues of the grafted peptide surfaced. Models were prepared with SWISS-MODEL server and figures with PyMOL (PyMOL Molecular Graphics System, Version 1.3 Schrödinger, LLC). (**b**) HPLC-SEC profiles of anti-hTfr peptide-engrafted versions of hCD81 LEL, from bottom to top: molecular weight standards at 670, 158, 44, 17 and 1.3 kDa (Bio-Rad), wild-type hCD81 LEL, hCD81 LEL_Tfr1, hCD81 LEL_Tfr2 and hCD81 LEL_Tfr2_C4. (**c**) Far-UV CD of wild-type hCD81 LEL (dashed line), hCD81 LEL C4 (full line), hCD81 LEL_Tfr1 (dotted line), hCD81 LEL_Tfr2 (dash-2 dots line), and hCD81 LEL_Tfr2_C4 (dash-dotted line). hTfr: human transferrin receptor.

First the hydrophobic patch of the unstructured C-terminal part of helix D was chosen for peptide grafting. The peptide sequence that acts as a ligand specific for hTfr, flanked with a short flexible linker sequence Ser-Ser-Gly, was inserted between the residues Ser179 and Cys190 (model in Figure 5a) to give a mutant hCD81 LEL_Tfr1. The resulting protein was purified at 24 mg per L ExpiCHO supernatant and was found to be monomeric in SEC-HPLC (Figure 5b). Next, the same binding sequence was grafted at the site usually containing a short hairpin loop (Asp137-Asp139) that connects helix A to helix B in hCD81 LEL to give the mutant hCD81 LEL_Tfr2. The yield of purified protein amounted to only 3 mg/L ExpiCHO supernatant and SEC analysis revealed a broad peak eluting at a later time point than wild-type hCD81 LEL, indicating non-specific interaction with the column matrix (Figure 5b). The most potent variant of mutants designed for a *de novo* cysteine bond that can interconnect helix A and helix B was then introduced as a stabilization motif into this peptide-grafted mutant to yield a protein termed hCD81 LEL_Tfr2_C4 (model in Figure 5a). The expression level of this protein increased to 10.5 mg/L and the SEC-HPLC profile was now similar to the wild-type protein (Figure 5b). The far-UV CD spectrum of hCD81 LEL_Tfr2_C4 was identical to the one of wild-type hCD81 LEL and the parental scaffold protein C4 (Figure 5c). For the unstabilized peptide-engrafted variant hCD81 LEL_Tfr2 only about 30% ellipticity was observed. Interestingly, the far-UV CD spectrum of hCD81 LEL_Tfr1 showed 13% less negative ellipticity at 222 nm, but a more prominent absorbance band shifted towards lower wavelengths from the 208-nm minimum, characteristic for spectra of other mutants.

### 3.5. Internalization of hTfr Reactive Peptide-Engrafted Mutants

**Figure 6:**
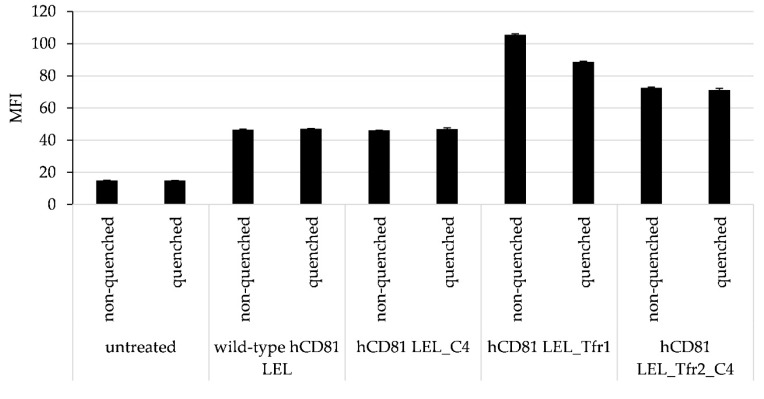
Enhanced internalization of anti-hTfr peptide-engrafted mutants of hCD81 LEL into the target-positive cell line SK-BR3.

The cell line SK-BR3 expressing high levels of hTfr [31] (verified by titration with the anti-hTfr antibody MEM-75 [32], Supplementary Figure S3), was used as a test cell line for internalization of peptide-engrafted mutants hCD81 LEL_Tfr1, hCD81 LEL_Tfr2, and the hCD81 LEL_Tfr2_C4 variant. First, we determined the hTfr receptor numbers on the cell surface to hCD81 LEL to be 356,480 ± 6943 copies per cell. Mutants engrafted with hTfr-cognate peptide sequence and the corresponding scaffold proteins were allowed to internalize into SK-BR3 cells. The level of internalization was determined by comparing the mean fluorescence values of the cell sample where the surface fluorescence was quenched using an anti-Alexafluor488 antibody and a non-quenched sample. After 12 h, the internalization of the variants engrafted with the hTfr-reactive peptide was significantly higher than was observed for the wild-type hCD81 LEL and its stabilized variant C4 (Figure 6).

## 4. Discussion

Extracellular domains of tetraspan proteins enriched in the membrane of extracellular vesicles harbor a potential for specific recognition and directional uptake of these mediators of cellular communication, which significantly influences cellular processes such as differentiation [33], aging [34], and cancer (reviewed in [35]). In order to generate functionalized surface of exosomal vesicles (EVs), we have here engineered CD81 as a model EV marker protein. We have aimed to design mutant proteins with a stabilized large extracellular loop of hCD81 using molecular modeling approach successfully applied previously for stability engineering of different proteins [36,37]. Indeed, we successfully identified stabilizing mutations beneficial to this particular folding unit. Its high expression level, monodisperse SEC profile, and its dimeric status indicative of correct tertiary structure [38] confirm its amenability as a standalone protein scaffold. The first site deemed suitable for peptide grafting is relatively unstructured in the parental molecule and could be engrafted with a 7-amino acid sequence N- and C-terminally encompassed with Ser-Ser-Gly flexible linker without prior stabilization of the folding motive. The assumption that this part of the molecule is amenable for mutagenesis was based on its low level of structural conservation among the members of the tetraspan family and on its hydrophobic features. As this region contains the binding site of the antibody M38, which reacts with cell-bound hCD81, we assumed its full accessibility for binding to the target antigen when presented on the cell surface in a full-length hCD81 context. Nevertheless, modifications of this site might influence the interaction of CD81 with other tetraspan molecules and the formation of the tetraspan web on the cell surface [20]. Further, the far-UV CD spectrum of hCD81 LEL_Tfr1 revealed an about 10% decrease in the ellipticity at 222 nm, which could indicate a partial loss of α-helical structure. The fact that the absorbance band at 208 nm was shifted towards a lower wavelength and more pronounced in the respect to the wild type could again originate from the loss of secondary structure, but could also be a consequence of absorbance of the newly introduced aromatic side chains in the sequence of the engrafted peptide. Therefore, it was attractive to test another solvent-exposed site for the peptide graft. We chose the short hairpin loop connecting helices A and B for grafting of the hTfr binding sequence. The protein modified in this way exhibited a strong interaction with the column matrix in SEC-HPLC in native conditions and a significantly lower ellipticity in far-UV CD, which could be a consequence of unfolding of the secondary structure or aggregation of the protein preparation. The application of such molecules in an in vivo setting is limited as unstructured protein strands are prone to serum protease-mediated degradation and other undesired effects such as aggregation. After the introduction of a *de novo* cysteine bond between helices A and B, the grafted mutant showed an elution profile as well as a far-UV CD spectrum indistinguishable from the wild-type hCD81 LEL. We assume that several of the identified stabilizing disulfide bonds could enhance the biophysical properties of the hCD81 LEL engrafted with a peptide sequence at this position, such as C3 and C7, both designed to interconnect helices A and B, and C9, connecting helix A with helix C. It would be very interesting to examine the resulting proteins for their folding and stability and relate their properties to the extent of stabilization that the proposed mutations confer to the wild-type molecule. Both peptide-engrafted variants of hCD81 LEL could internalize into the strongly target-positive cell line SK-BR3 more efficiently than their wild-type scaffold counterparts.

As several disulfide bonds that interconnect topologically different parts of the molecule were identified, a mutant with two combined *de novo* disulfide bridges was examined and shown to display potentiated stability. Such superior versions of the hCD81 LEL are expected to be permissive of randomization in various regions of the original fold. Particularly unexpected was the discovery of mutant Ala130Cys/Val146Cys, which exhibited reversible thermal unfolding even when heated up to 110 °C. Reversible unfolding of a eukaryotic multispan membrane protein was described before [38] and its stability in micelles was found to be dependent on the presence of “chemical chaperones”.

Ligand–receptor interactions on the recipient cell surface have been studied extensively in the biology of uptake of exosomes. Several characteristic membrane exosome proteins, including CD81, have been shown to participate in the cellular uptake of exosomes as ligand proteins. Traditionally, endocytosis was believed to be the major pathway for the cellular uptake of ligands, plasma membrane proteins, and lipids; however, other mechanisms of their efficient cellular entry have been elucidated [39,40]. While clathrin- or caveolin-mediated endocytosis is of limited efficiency for particles of 100 nm in size, which is typical for exosomes, larger volumes of liquid can be taken into the cell by micropinocytosis [41]. The mechanism of uptake may influence tissue specificity of exosomal ingestion, but the cellular fate is as well dependent on the pathway of cellular trafficking [42]. When engulfed into macropinosomes that do not fuse with lysosomes, exosomal contents might be protected from detrimental digestion before their release to the extracellular fluid by recycling. Therefore it would be extremely interesting to directionally engineer CD81 to assist the cellular uptake of exosomes through the macropinocytosis pathway, induced by stimulation of macropinocytosis-related receptors and the oncogenic Ras-protein. We believe that the engagement of the receptors dedicated to mediate this pathway can be achieved by variants of the scaffold protein stabilized to sustain perturbations caused by mutagenesis, which can be decisive for the successful therapeutic application of EVs derivatized in this way.

## Figures and Tables

**Table 1:** Variants of human CD81 large extracellular loop (hCD81 LEL) designed for stabilization with **de novo** cysteine bonds. Indicated are amino acids that were mutated to cysteine, their position and the measured melting temperature (*T*_m_).

hCD81 LEL	Mutated Position 1		Mutated Position 2		*T*_m_ (°C)
Variant	Located on Segment	Amino Acid	Located on Segment	Amino Acid	
wild type					66.15 ± 0.25
C1	Helix A	Ala134	Helix B	Ala143	n.d. ^1^
C2	Helix A	Ala130	Helix B	Val146	67.35 ± 0.05
C3	Helix A	Gln133	Helix B	Ala143	82.15 ± 0.05
C4	Helix A	Ala134	Helix B	Lys144	88.95 ± 0.05
C5	Helix B	Leu154	Helix E	Lys193	n.d.
C6	Helix A	Ala120	Helix E	Phe198	n.d.
C7	Helix A	Ala130	Helix B	Ala143	76.90 ± 0.00
C8	Helix A	Leu131	Helix C	Leu165	n.d.
C9	Helix A	Val135	Helix C	Ser168	90.45 ± 0.15
C10	Helix C	Val169	Unstructured part of segment C	Leu174	n.d.
C11	Loop preceeding helix C	Ser160	Start of helix D	Asp189	70.25 ± 0.15

^1^ n.d., not determined.

## Supplementary Materials

The following are available online at http://www.mdpi.com/1999-4923/10/3/138/s1, Figure S1: ELISA testing the reactivity of *de novo* disulfide bond endowed hCD81 LEL mutants with structure-reporter antibody M38; Figure S2: SDS-PAGE of purified hCD81 LEL variants with *de novo* disulfide bonds; Figure S3: Titration of binding of anti-hTfr antibody MEM-75 to the surface of SK-BR3 cells; Table S1: Amino acid sequences of hCD81 LEL and peptide-engrafted proteins; Table S2: Oligonucleotides used for the construction of *de novo* disulfide bond-stabilized mutants.
