# Supplementary material for: Stabilization of the CD81 Large Extracellular Loop with De Novo Disulfide Bonds Improves Its Amenability for Peptide Grafting
Source: Pharmaceutics. 2018 Aug 27;10(3):138. doi: 10.3390/pharmaceutics10030138 (PMC6160918; doi:10.3390/pharmaceutics10030138)
Supplement: Supplementary file 1 [file pharmaceutics-10-00138-s001.pdf]

# Supplementary Materials: Stabilization of the CD81 Large Extracellular Loop with *De Novo* Disulfide Bonds Improves Its Amenability for Peptide Grafting

**Stefan Vogt, Gerhard Stadlmayr, Katharina Stadlbauer, Flávio Sádio, Peter Andorfer, Johannes Grillari, Florian Rüker and Gordana Wozniak-Knopp**

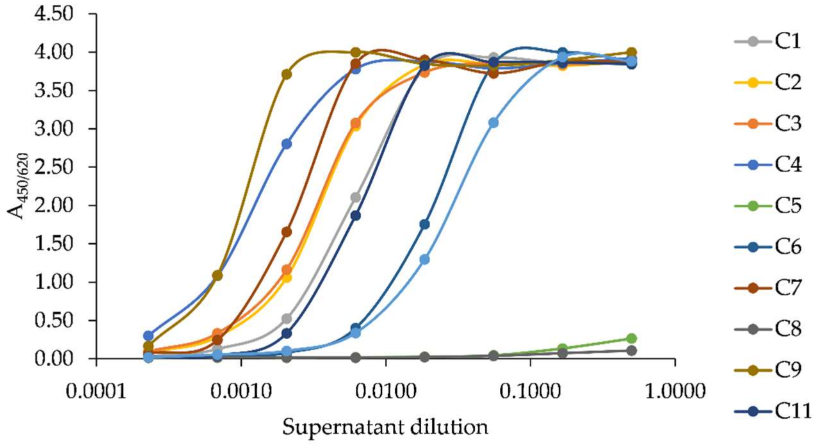


**Figure S1.** ELISA testing the reactivity of *de novo* disulfide bond endowed hCD81 LEL mutants with structure-reporter antibody M38.

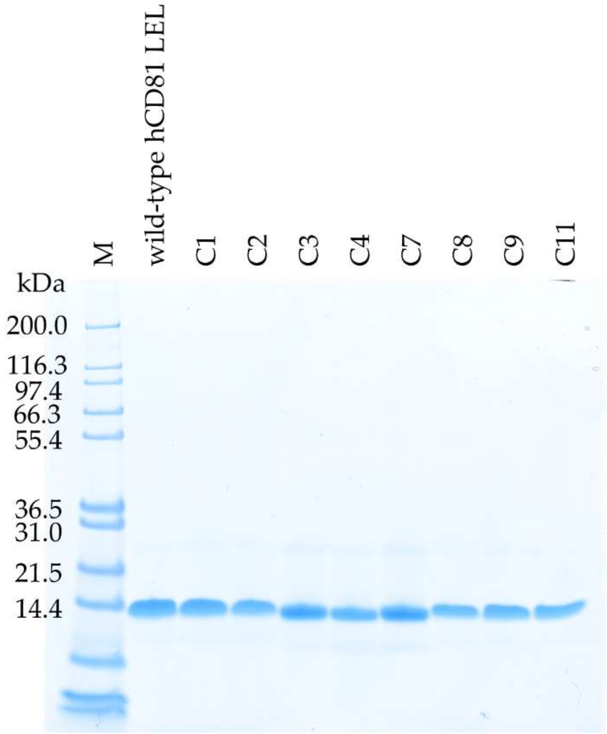


**Figure S2.** SDS-PAGE of purified hCD81 LEL variants with *de novo* disulfide bonds.

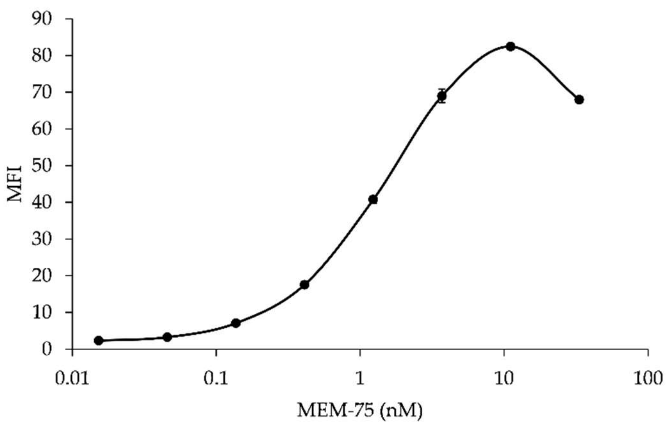


**Figure S3.** Titration of binding of anti-hTfr antibody MEM-75 to the surface of SK-BR3 cells.

**Table S1.** Amino acid sequences of hCD81 LEL and peptide-engrafted variants. First and last residue pertaining to hCD81 LEL sequence are in bold, native cysteine residues are in orange, the sequence of peptide graft in in blue and novel cysteine residues are in green, his-tag sequence is underlined.

| **hCD81 LEL Mutant** | **Amino Acid Sequence** |
|---|---|
| wild-type | <u>HHHHHHHH</u>GSTGENLY**F**QGASFVNKDQIAKDVKQFYDQALQQAVVDDDANNAKAVVKTFHETLDCCGSSTLTALTTSVLKNNLCPSGSNIISNLFKEDCHQKIDDLFSG**K** |
| hCD81 LEL_Tfr1 | <u>HHHHHHHH</u>GSTGENLY**F**QGASFVNKDQIAKDVKQFYDQALQQAVVDDDANNAKAVVKTFHETLDCCGSSTLTALTTSVLKNNLCPSGSN**HAIYPRHSSG**CHQKIDDLFSG**K** |
| hCD81 LEL_Tfr2 | <u>HHHHHHHH</u>GSTGENLY**F**QGASFVNKDQIAKDVKQFYDQALQQAVV**GSSHAIYPRHSSG**ANNAKAVVKTFHETLDCCGSSTLTALTTSVLKNNLCPSGSNIISNLFKEDCHQKIDDLFSG**K** |
| hCD81 LEL_Tfr2_C4 | <u>HHHHHHHH</u>GSTGENLY**F**QGASFVNKDQIAKDVKQFYDQALQQCVV**GSSHAIYPRHSSG**ANNACAVVKTFHETLDCCGSSTLTALTTSVLKNNLCPSGSNIISNLFKEDCHQKIDDLFSG**K** |

**Table S2.** Oligonucleotides used for the construction of *de novo* disulfide bond stabilized mutants.

| hCD81 LEL Variant | Substitution | Oligonucleotide Sequence |
|---|---|---|
| C1 | Ala134Cys | CAGGCCCTACAGCAGTGCGTGGTGGATGATGAC<br>GTCATCATCCACCACGCACTGCTGTAGGGCCTG |
| | Ala143 Cys | GATGATGCCAACAACTGCAAGGCTGTGGTGAAG<br>CTTCACCACAGCCTTGCAGTTGTTGGCATCATC |
| C2 | Ala130Cys | CAGTTCTATGACCAGTGTCTACAGCAGGCCGTG<br>CACGGCCTGCTGTAGACACTGGTCATAGAACTG |
| | Val146Cys | AACAACGCCAAGGCTTGTGTGAAGACCTTCCAC<br>GTGGAAGGTCTTCACACAAGCCTTGGCGTTGTT |
| C3 | Gln133Cys | GACCAGGCCCTACAGTGCGCCGTGGTGGATGATG<br>CATCATCCACCACGGCGCACTGTAGGGCCTGGTC |
| | Ala143Cys | GATGATGCCAACAACTGCAAGGCTGTGGTGAAG<br>CTTCACCACAGCCTTGCAGTTGTTGGCATCATC |
| C4 | Ala134Cys | CAGGCCCTACAGCAGTGCGTGGTGGATGATGAC<br>GTCATCATCCACCACGCACTGCTGTAGGGCCTG |
| | Lys144Cys | GACGCCAACAACGCCTGTGCTGTGGTGAAGACC<br>GGTCTTCACCACAGCACAGGCGTTGTTGGCGTC |
| C5 | Leu154Cys | GACCTTCCACGAGACGTGTGACTGCTGTGGCTC<br>GAGCCACAGCAGTCACACGTCTCGTGGAAGGTC |
| | Lys193Cys | GAGGACTGCCACCAGTGTATCGATGACCTCTTC<br>GAAGAGGTCATCGATACACTGGTGGCAGTCCTC |
| C6 | Ala120Cys | CAACAAGGACCAGATCTGTAAGGATGTGAAGCAG<br>CTGCTTCACATCCTTACAGATCTGGTCCTTGTTG |
| | Phe198Cys | AAGATCGATGACCTCTGTTCCGGGAAGTGATGAG<br>CTCATCACTTCCCGGAACAGAGGTCATCGATCTT |
| C7 | Ala130Cys | CAGTTCTATGACCAGTGTCTACAGCAGGCCGTG<br>CACGGCCTGCTGTAGACACTGGTCATAGAACTG |
| | Ala143Cys | GATGATGCCAACAACTGCAAGGCTGTGGTGAAG<br>CTTCACCACAGCCTTGCAGTTGTTGGCATCATC |
| C8 | Leu131Cys | TTCTATGACCAGGCCTGCCAGCAGGCCGTGGTG<br>CACCACGGCCTGCTGGCAGGCCTGGTCATAGAA |
| | Leu165Cys | AGCACACTGACTGCTTGTACCACCTCAGTGCTC<br>GAGCACTGAGGTGGTACAAGCAGTCAGTGTGCT |
| C9 | Val135Cys | GCCCTACAGCAGGCCTGTGTGGATGATGACGCC<br>GGCGTCATCATCCACACAGGCCTGCTGTAGGGC |
| | Ser168Cys | GCTTTGACCACCTGTGTGCTCAAGAACAAT<br>ATTGTTCTTGAGCACACAGGTGGTCAAAGC |
| C10 | Val169Cys | TTGACCACCTCAGTGTGCAAGAACAATTTGTGTC<br>GACACAAATTGTTCTTGCACACTGAGGTGGTCAA |
| | Leu174Cys | GTGCTCAAGAACAATTGTTGTCCCTCGGGCAGC<br>GCTGCCCGAGGGACAACAATTGTTCTTGAGCAC |
| C11 | Ser160Cys | GACTGCTGTGGCTCCTGTACACTGACTGCTTTG<br>CAAAGCAGTCAGTGTACAGGAGCCACAGCAGTC |
| | Asp189Cys | AACCTCTTCAAGGAGTGTTGCCACCAGAAGATC<br>GATCTTCTGGTGGCAACACTCCTTGAAGAGGTT |
